# Distribution of ABO and Rh-D blood groups in the Benin area of Niger-Delta: Implication for regional blood transfusion

**DOI:** 10.4103/0973-6247.39502

**Published:** 2008-01

**Authors:** Mathew Ebose Enosolease, Godwin Nosa Bazuaye

**Affiliations:** *Department of Haematology, University of Benin Teaching Hospital, Benin City, Nigeria*

**Keywords:** ABO, Rh-D, Niger-Delta

## Abstract

ABO and Rhesus (Rh) blood group antigens are hereditary characters and are useful in population genetic studies, in resolving medico-legal issues and more importantly in compatibility test in blood transfusion practice. Data on frequency distribution of ABO and Rh-D in Niger-Delta region of Nigeria are not available; hence we made an attempt to retrospectively analyze the records on the blood donors, transfusion recipients and patients attending antenatal care or some other medical interventions. Over a twenty-year period between 1986 and 2005, a total of 160,431 blood samples were grouped for ABO and Rh-D at the blood bank of the University of Benin Teaching Hospital, Benin City, Nigeria. Blood group distribution among these samples showed phenotypes A, B, AB and O as 23.72%, 20.09%, 2.97% and 53.22%, respectively. The Rh-D negative phenotype was found among 6.01% of the samples tested.

## Introduction

The ABO blood groups and Rhesus (Rh) blood group antigens are the most frequently studied genetic markers in a large group of people.[1] Despite the long list of several other blood groups discovered so far, the ABO blood groups hold a respectable position in view of the safety of blood/blood products transfusion to date.[2] The knowledge of the distribution of ABO and Rh blood groups is essential for effective management of blood banks inventory, be it a facility of a smaller local transfusion service or a regional or national transfusion service.

Apart from their importance in blood transfusion practice, the ABO and Rh blood groups are useful in population genetic studies, researching population migration patterns, as well as resolving certain medico-legal issues, particularly of disputed parentage. It is, therefore, imperative to have information on the distribution of these blood groups in any population group. A limited study investigated the ABO blood group frequency in certain population in and around Ibadan in Western Nigeria.[1,3]

The population structure of Benin City, with over 9 million people, mainly comprises ethnic groups including: the Binis, Esans, Afemai, Urhobos, Ijaws, Ibos of the Delta and the Yorubas in Akoko-Edo. The collation of immunohaematology data would, therefore, enhance sustainable regional blood bank services in the region of Niger-Delta. This study seeks to provide data on ABO and Rh-D blood group distribution in the Benin region of Niger-Delta.

## Materials and Methods

### Population and period

Records of blood groupings of blood donors, blood recipients, patients attending routine antenatal care as well as individuals who presented for routine medical examinations between 1986 and 2005 were examined, and care was exercised to eliminate any repeat entry. All entries were double-checked by each author.

ABO and Rh blood groupings were carried out in our blood bank by standard tile techniques with appropriate positive and negative controls using one drop of whole blood mixed with one drop of appropriate anti-sera and rocked gently. In case of doubt, the test was examined under a microscope, or the results were confirmed by reverse grouping using known group A and B red cells (Dacie and Lewis).[4] Data on the frequency of ABO and Rh-D blood groups were reported in simple percentages.

## Results

During the period between 1986 and 2005, a total of 160,431 blood samples were collected. Of these, 71,506 (44.57%) were from blood donors, 50,100 (31.23%) were from transfusion recipients, 31,009 (19.33%) were from antenatal cases and 7,916 (4.87%) were from routine medical sampling.

Table 1 shows the distribution of various ABO and Rh-D phenotypes among the samples studied. The frequency of group O phenotype comprised over half (53.22%) the samples, while group AB was the least encountered phenotype with a frequency of less than 3% among the samples studied. The frequency of groups A and B accounted for 23.72% and 20.09%, respectively.

**Table 1:** The frequency of blood groups ABO and Rh-D phenotypes in the population sample obtained from different sources

Blood samples	ABO blood group phenotypes				Rh-D phenotypes	
		
	A	B	AB	O	Rh-D positive	Rh-D negative
Blood donors	17,018 (23.80)	14,801 (20.70)	2,002 (2.80)	37,685 (52.70)	67,128 (93.88)	4,378 (6.12)
Recipients	11,674 (23.30)	10,020 (20.00)	1,504 (3.00)	26,902 (53.70)	47,099 (94.01)	3,001 (5.99)
Antenatal	7,567 (22.99)	5,859 (18.89)	1,024 (3.30)	16,559 (53.40)	29,190 (94.13)	1,819 (5.94)
Routine medical examination	1,797 (22.99)	1,555 (19.90)	234 (2.99)	4,230 (54.12)	7,352 (94.06)	4,64 (5.94)
Total	38,056 (23.72)	32,235 (20.09)	4,764 (2.97)	85,376 (53.22)	67,128 (93.88)	9,674 (6.03)

*P*-values for blood groups A, B, AB and O were 0.999, 0.980, 0.942 and 0.745, respectively, among the different sources of blood samples; Figures in parentheses are in percentage

Rh-D antigen was detected in 67,128 (93.88%) samples while Rh-D negative phenotype was found in 9,674 (6.03%) samples in the total sample size of 160,431.

Apparently, there was difference in the distribution of ABO and Rh-D phenotypes among the samples obtained from different sources.

Table 2 shows the frequency of Rh-D phenotype among the samples studied with respect to the ABO blood groups. Rh-D negative was common to the blood group ‘O’ (3% of all blood group ‘O’) as compared to blood group AB of only 0.19%. The Rh group negativity for blood groups A and B were close (1.58% and 1.26%, respectively). There was no association between Rh status and ABO blood group. The frequency of Rh (Rh-D) is shown in Table 1. Moreover, the source of blood has no influence on the Rh status, *P* = 0.824 (χ^2^ = 0.049).

**Table 2:** The frequency of Rh-D in the various ABO blood groups of the different population samples

Sources	A+	A−	B+	B−	AB+	AB−	O+	O−	Total
Blood donors	15,844 (22.16)	1,174 (1.64)	13,868 (19.39)	933 (1.30)	1,880 (2.63)	122 (0.22)	35,536 (49.69)	2,149 (3.01)	71,506 (44.57)
Recipients	10,895 (21.75)	778 (1.55)	9,391 (18.74)	629 (1.26)	1,410 (2.81)	94 (0.18)	25,383 (50.66)	1,519 (3.03)	50,100 (31.23)
Antenatal	7,090 (22.86)	477 (1.54)	5,495 (17.72)	364 (1.23)	956 (3.08)	68 (0.18)	15,649 (50.49)	910 (2.93)	31,009 (19.33)
Routine examination	1,690 (21.62)	107 (1.37)	1,459 (18.67)	96 (1.23)	220 (2.81)	14 (0.18)	3,983 (50.96)	247 (3.16)	7,816 (4.87)
Total	35,520 (22.14)	2,536 (1.58)	30,213 (18.83)	2,022 (1.26)	4,466 (2.78)	298 (0.19)	80,551 (50.21)	4,825 (3.01)	160,431 (100)

A+, *P* = 0.546; A−, *P* = 0.293; B+, *P* = 0.581; B−, *P* = 0.900; AB+, *P* = 0.877; AB−, *P* = 0.832; O+, *P* = 0.317; O−, *P* = 0.510%; Figures in parentheses are in percentage

The frequency of A, B, AB and O and Rh-D blood groups from different sample populations were assumed as raw data for the purpose of Chi-square calculation. The association between source of blood and blood group was tested using Chi-square test. The level of significance was set at a probability of 5%. All calculations were done using Graph-Pad Instant™ (1994).

Wolledge *et al*. and Bakare *et al*. reported A = 24.9, among the Yurobas at Ibadan (Western Nigeria) while Bakare *et al*. reported A = 22.9%, B = 21.3%, AB = 5.9%, O = 50% and 3.3% Rh-D negative among the Yurobas at Ogbomosho, which is less than 100 km away from Ibadan.[1,4] This disparity was instructive even among the same ethnic group. Mourant *et al*. have earlier shown that the frequency of ABO/Rh blood group is valid only for the specific region or the specific population group from where the data were derived.[8]

## Discussion

ABO and Rh genes and phenotypes vary widely across races and geographical boundaries[4–6] despite the fact that the antigens involved are stable throughout life. The resultant polymorphism remains important in population genetic studies, estimating the availability of compatible blood, evaluating the probability of haemolytic disease in the new born, resolving disputes in paternity/maternity and for forensic purposes.[2,7] The present study is, therefore, useful in providing information on the status of ABO and Rh blood group distribution in the Niger-Delta region of Nigeria.

Insofar the distribution of ABO blood groups is concerned, the group ‘O’ (53.22%) is the most frequently encountered phenotype in the population under study. This observation is in accordance with previous reports from other parts of Nigeria. In Southern Nigeria,[1] Binis and Yorubas had group ‘O’ frequency of 57.7% and 51.1%, respectively, while in the northern part[8] it was reported as 52%.

With regard to the other phenotypes of ABO blood groups, the frequency of group ‘A’ as 23.72% in the present study is in proximity to the reported frequency of 24.9% for group ‘A’ among the Binis.[1] A slightly higher frequency for group A (27%) was reported in the north-east region of Nigeria.[8] A lower proportion of group ‘B’ as 14.5% and in the northern regions in comparison to the present study appears to be a compensatory effect created by a slightly higher frequency of group ‘O’ in the Binis.[1] The discrepancy between our findings and that reported by Worlledge *et al*.[1] may be attributed to the ethnic difference among the population of Nigeria, or it could be due to the smaller sample size of the Binis as against a relatively larger sample size in the present study. The latter view has grounds in the report on the Yorubas,[1] which was based on a large series and in accordance with the frequency for groups ‘A’ and ‘B’ in the present study (in the Yorubas, the frequency of groups ‘A’ and ‘B’ was reported as 21.3% and 23.3%, respectively).

A similarity in the distribution of ABO phenotypes reported in North American Blacks[9] with that of the Nigerian population may indicate influencing factors such as genetic drift and ancestral link with the people of Niger-Delta through the Trans-Atlantic trade because of the proximity to ocean between these countries.

The present study shows that the frequency of Rh-D negative phenotype is close to those reported among the Yorubas (5.46%) but differs greatly than that reported from north-eastern Nigeria (1.44%). Moreover, this shows ethnic variability among the Nigerians. A reported frequency of around 5% of Rh-D negative phenotype from India is close to the one reported in the present series and in sharp contrast to the frequency of 15% Rh-D negative phenotype reported in the people of European origin.[6,10,11]

## Conclusion

With a large sample, we established that among the various ABO and Rh-D blood groups in the Niger-Delta of Nigeria, group ‘O’ is the commonest, and the occurrence of blood groups ‘A’ and ‘B’ is nearly equal; the frequency of Rh-negative is, although slightly higher in the present series than that in the previous studies in other parts of Nigeria, was strikingly lower than the Caucasian population.
